# A novel pathogenic mutation of the CYP27B1 gene in a patient with vitamin D-dependent rickets type 1: a case report

**DOI:** 10.1186/1756-0500-7-783

**Published:** 2014-11-05

**Authors:** Amir MI Babiker, Iman Al Gadi, Nasir AM Al-Jurayyan, Abdulrahman MH Al Nemri, Ali Abdu N Al haboob, Ahmed Amer Al Boukai, Ali Al Zahrani, Hanan Ahmed Habib

**Affiliations:** Pediatric Department, College of Medicine and King Khalid University Hospital, Riyadh, Saudi Arabia; Pediatric Endocrine Division, King Khalid University Hospital and King Saud University, Riyadh, Saudi Arabia; Radiology and Medical Imaging Department, King Khalid University Hospital and King Saud University, Riyadh, Saudi Arabia; Pathology Department, King Khalid University Hospital and King Saud University, Riyadh, Saudi Arabia; Pediatric Department, College of Medicine, King Khalid University Hospital and King Saud University, PO Box 2925, Riyadh, 11461 Saudi Arabia

**Keywords:** CYP27B1, 1,25 dihydroxyvitamin D3, Missense mutation, Rickets, Saudi Arabia, VDDR-1

## Abstract

**Background:**

Rickets can occur due to Vitamin D deficiency or defects in its metabolism. Three rare genetic types of rickets with different alterations of genes have been reported, including: Vitamin D dependent rickets type 1, Vitamin D dependent rickets type 2 or also known as Vitamin D resistant rickets and 25 hydroxylase deficiency rickets. Vitamin D dependent rickets type 1 is inherited in an autosomal recessive pattern, and is caused by mutations in the CYP27B1 gene encoding the 1α-hydroxylase enzyme. We report here a new mutation in CYP27B1, which lead to Vitamin D dependent rickets type 1.

**Case presentation:**

We report on a 13-month-old Arabic Saudi girl with Vitamin D dependent rickets type 1 presented with multiple fractures and classic features of rickets. A whole exome sequencing identified a novel pathogenic missense mutation (CYP27B1:Homozygous c.1510C > T(p.Q504X)) which results in a protein truncating alteration. Both parents are heterozygous carriers of the mutation. Based on data search in Human Gene Mutation Database, 63 CYP27B1 alterations were reported: only 28.6% are protein truncating (5 nonsense, 13 frameshift insertions/deletions, 0 gross deletions), while 61.9% are non-truncating (38 missense, 1 small in-frame insertions/deletion), and 9.5% are possible protein-truncating (5 splice, 1 regulatory).

**Conclusion:**

The deleterious effect of this alteration, which was the only mutation detected in the CYP27B1 common gene of Vitamin D dependent rickets type 1 in the proband, and its autosomal recessive inheritance fashion, both support a pathogenic nature of this mutation as the cause of Vitamin D dependent rickets type 1.

## Background

**Figure 1:**
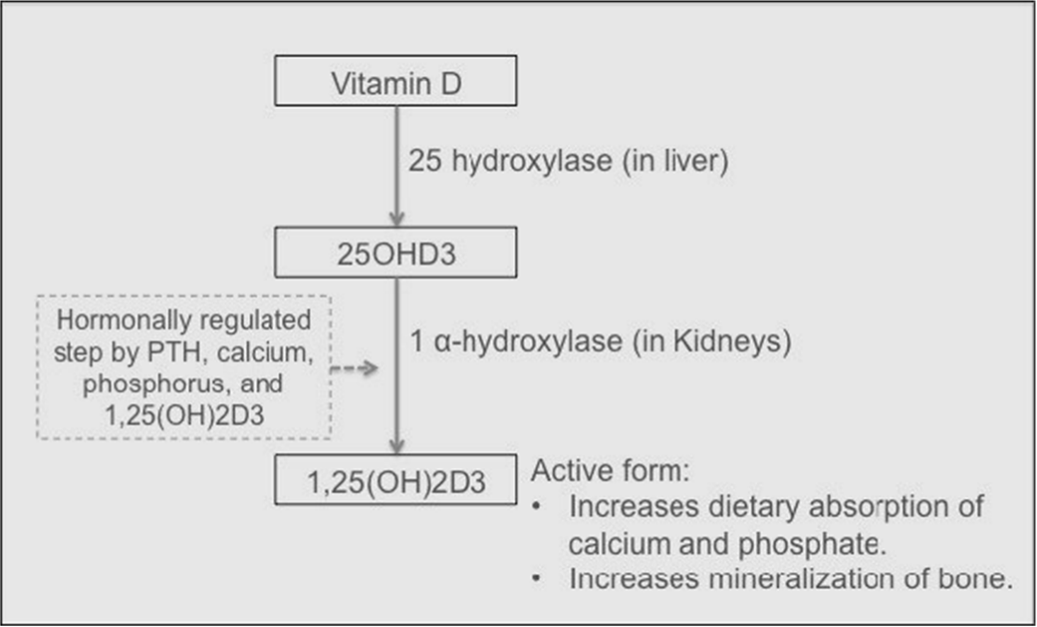
**Bio-activation pathway of vitamin D.**

Vitamin D, whether synthesized in the skin or obtained from the diet, is biologically inert and requires activation via two selective metabolic steps; the second of them is rate-limiting and hormonally regulated (Figure 1). Both of the steps are catalyzed by cytochrome P450 enzymes to form the active secosteroid hormone 1,25 dihydroxyvitamin D3 (1,25(OH)_2_D3) [1, 2].

Vitamin D deficiency remains the commonest cause of rickets worldwide [3, 4]. So far, apart from liver or renal diseases that may affect vitamin D metabolism, three rare genetic errors of vitamin D metabolism that can cause rickets have been described [3–6]. The first involves 1α-hydroxylase deficiency, also described as vitamin D dependent rickets type 1 (VDDR-1). The second type involves defective vitamin D receptor (VDR), resulting in vitamin D resistant rickets (VDRR), also known as vitamin D dependent rickets type II (VDDR II) [3]. More rare, 25 hydroxy vitamin D3 (25OHD3) deficiency have been reported and linked to a selective mutation in CYP2R1 gene that leads to 25-hydroxylase deficiency [4, 5].

Children with VDDR-1 may present with hypotonia, muscle weakness, joint pain and deformity, growth failure, seizures, or fractures in early infancy [3]. We report a 13-month-old girl with VDDR-1 presented with multiple fractures, classic biochemical and severe radiological findings of rickets. An initial CYP27B1 gene analysis for common alterations was performed and showed no disease-associated mutations in this patient. However, the whole exome sequencing identified a novel pathogenic mutation (CYP27B1: Homozygous c.1510C > T(p.Q504X)) in our patient.

## Case presentation

**Figure 2:**
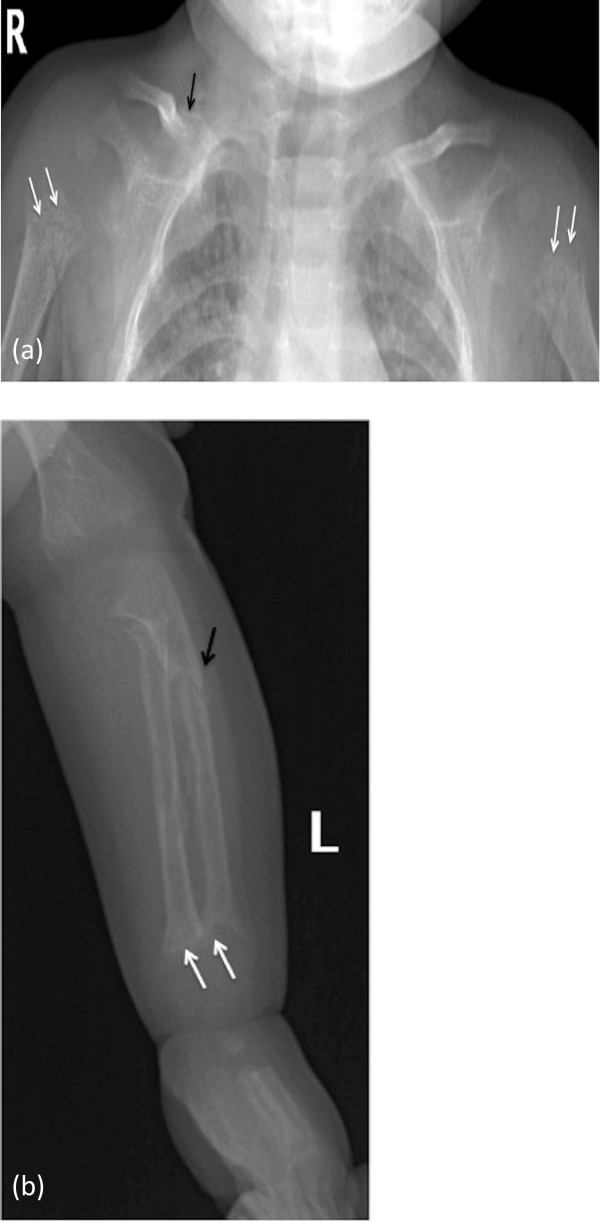
**X-ray images at time of presentation showing generalized osteopenia with altered texture (a & b).** Humeral metaphysis shows frying with more pronounced lucency **(a)**, and ulnar/radial metaphysis shows frying and cupping of their outline **(b)** - (white arrows). The cortices are indistinct with coarse fuzzy trabecuale **(a & b)**. Fracture of right clavicle **(a)**, and proximal shaft of the ulna **(b)** - (black arrows).

A 13-month-old Arabic Saudi girl, previously well, presented with multiple fractures as a result of minor traumas. She was adequately exposed to sunlight and had normal perinatal, developmental, and nutritional history. She is the only child for first-cousins parents, with no family history of similar problems or metabolic bone disease. Examination revealed normal growth for age, mildly reduced muscle tone, normal teeth, rachitic rosary, Harrison’s sulcus, widening of the wrist and bowing deformity of distal arms and legs. Her head circumference was on the 50th centile for age with a frontal bossing and wide anterior fontanel. She had a swelling, secondary to fracture, over her right clavicle. However, the rest of her skin examination was normal and no other signs, which might suggest physical abuse. Initial investigations showed: low serum calcium and phosphorus, high serum parathyroid hormone (PTH), high serum alkaline phosphatase, a normal serum 25OHD3 which was repeated two times, and inappropriately low normal serum 1,25(OH)_2_D3 suggesting 1α-hydroxylation defect (Table 1). Radiological workup showed generalized osteopenia with multiple fractures involving the right clavicle, right ulna, left proximal ulna and tibia bones. Disorganization of the growth plate was apparent with classic features of rickets (Figure 2).

**Table 1:** **Biochemical laboratory results of the patient in relation to time and treatment**

Time (At presentation and afterwards)	Serum 25OHD3 [N = 75–250 nmol/L]	Serum 1,25(OH) _2_D3 [N = 15-75 pg/mL]	Serum PTH [N =1.65–6.9 pmol/L]	Serum Calcium [N = 2.25–2.75 mmol/L]	Serum Phosphorus [N = 1.45–2.16 mmol/L]	Serum Alkaline phosphatase [N = 175–476 IU/L]	Serum Magnesium [N = 0.7–1.1 mmol/L]
**At presentation**	119.5	16	52.9	1.94	0.82	1682	0.7
	**Treatment:** She was started with high-dose 1α-hydroxyvitamin D3 (2000 IU/day) and elemental calcium (61 mg/kg/day), it was gradually increased to 150 mg/kg/day with monitoring of serum levels						
**1 month**	99.9	-	54.9	1.90	0.86	2027	-
	**Treatment:** Addition of oral phosphate supplements. She developed diarrhoea, so it was stopped after 1 week						
**2 months**	99.3	-	55.4	1.6	0.90	1276	-
	**Treatment:** Continued on 1α-hydroxyvitamin D3 (2000 IU/day) and high-dose of calcium supplements (up to 200 mg/kg/day)						
**3 months**	-	-	Initially 14.00 then back to 21.36	2.06	1.02	1358	-
	**Treatment:** Started to wean off 1α-hydroxyvitamin D3. The PTH went high again, so it was initial dose was resumed again (2000 IU/day).						
**6 months**	-	-	21.41	2.19	1.41	503	-
**9 months**	-	-	0.97	2.44	1.94	233	-
	**Treatment:** 1α-hydroxyvitamin D3 was weaned off gradually down to (1200 IU/day), with calcium supplements (100 mg/kg/day)						
**10 months**	-	-	0.63	2.29	2.11	222	0.7
	**Treatment:** Daily Doses settled on 1α-hydroxyvitamin D3 (800 IU/day) and Calcium supplements (80 mg/kg/day)						

1,25(OH)2D3- 1,25 dihydroxyvitamin D3, 25OHD3- 25-hydroxyvitamin D3, and PTH- Parathyroid hormone.

In the light of the above biochemical results and severe radiological findings, genetic types of rickets were suspected. Initially, CYP27B1 gene analysis was performed only for the common mutations in exon 2 and 8 and it was negative. Unfortunately, the genetic laboratory, where the common mutations of CYP27B1 gene tests were tested, did not offer further analysis of the same common gene. Alternatively, It was suggested to perform mutations analysis for hypophosphataemic rickets but this was clinically and biochemically irrelevant in this patient. With a high clinical suspicion of 1α-hydroxylation defect, a whole exome sequencing test was performed in another genetic lab, with a report of a likely diagnosis of VDDR-1 (MIM-609506). The exome sequencing revealed a novel pathogenic nonsense deleterious mutation in the proband’s CYP27B1 gene (CYP27B1: Homozygous *c.1510C > T* (p.Q504X)), with both parents found to be heterozygous carriers of the mutation (Table 2).

**Table 2:** **Familial co-segregation analysis of the proband**

Gene(s)	Filtering model	Location of alteration	Alteration	Proband	Mother	Father
CYP27B1	Autosomal recessive	12q13.1-q13.3	c.1510C > T(p.Q504X)	Homozygous	Heterozygous	Heterozygous

Awaiting the genetic results, the patient required high doses of 1α-hydroxyvitamin D and calcium supplements to heal her bones and to return serum chemistries to normal (Table 1).

### Discussion

CYP27B1 gene is located on chromosome 12q13.3. It encodes a member of the cytochrome P450 superfamily of enzymes, which are monooxygenases that catalyze many reactions involved in drug metabolism and synthesis of cholesterol, steroids and other lipids. CYP27B1 gene encodes 1α-hydroxylase enzyme that is localized to the inner mitochondrial membrane of renal cells; in which it activates 25OHD3 to synthesise 1,25(OH)_2_D3, which binds to vitamin D receptor and regulates calcium metabolism. Homozygous or compound heterozygous alterations in this gene are inherited in autosomal recessive pattern, and are in association with VDDR-1 [7].

In our patient, the clinical, biochemical and radiological findings were all consistent with those of previously reported patients with CYP27B1 gene alterations [3, 8]. The first reported negative result of genetic mutations suggestive of VDDR-1 was based on inadequate examination, only for the common alterations in exons 2 and 8 of CYP27B1. This, beside the suggestion of further testing for hypophosphataemic rickets by the genetic lab, which was clinically irrelevant in our patient, both had mislead the directions of choice for further genetic testing. It would have been reasonable to perform a full analysis of the CYP27B1 gene alone for any other mutations in the first place, which could have detect the novel mutation in our patient, before proceeding for a whole exome sequencing in this patient. Nonetheless, genetic analysis with full exome sequencing, bioinformatics analysis and filtering based on autosomal and X-linked dominant and recessive inheritance models of the proband, mother, and father revealed 53 genes (70 unique alterations) (Table 3). Manual review to rule out sequencing artefacts and polymorphisms along with the medical interpretation to rule out genes lacking clinical overlap with the patient’s evaluated phenotype resulted in 25 genes (36 unique alterations) (Table 3).

**Table 3:** **Full exome-sequencing results of the patient**

Variant filtering based on inheritance model, clinical and bioinformatics analysis						
	Post-inheritance model filtering	Post-alteration review	Post-medical review			Candidate genes
		Total	Post-clinical associated review			
			Characterized	Clinically novel	Total	
**Autosomal dominant genes (alterations)**	12(13)	2(2)	0(0)	1(1)	1(1)	0(0)
**Autosomal recessive genes (alterations)**	41 (57)	32(45)	1(1)	23(34)	24(35)	1(1)
**X-linked recessive genes (alterations)**	0(0)	0(0)	0(0)	0(0)	0(0)	0(0)
**X-linked dominant genes (alterations)**	0(0)	0(0)	0(0)	0(0)	0(0)	0(0)
**Y-linked genes (alterations)**	N/A	N/A	N/A	N/A	N/A	N/A
**Total genes (alterations)**	53(70)	34(47)	1(1)	24(35)	25(36)	1*(1)

*The candidate gene in this patient is CYP27B1 gene, which is located in chromosome 12q13.3 and encodes a member of the cytochrome P450 superfamily of enzymes. The cytochrome P450 proteins are monooxygenases, which catalyze many reactions involved in drug metabolism and synthesis of cholesterol, steroids and other lipids. The protein encoded by this gene localizes to inner mitochondrial membrane where it hydroxylates 25-hydroxyvitamin D3 at the 1 alpha position. This reaction synthesizes 1, 25 dihydroxyvitamin D3, which binds to vitamin D receptor and regulates calcium metabolism. Thus this enzyme regulates the level of biologically active vitamin D and plays a major role in calcium homeostasis. Mutations in this gene can result in vitamin D dependent rickets type 1.

Alterations with likely clinical relevance “candidate genes” underwent co-segregation analysis, in which 1 candidate gene (1 alteration): (CYP27B1:Homozygous *c.1510C > T*(p.Q504X)) was selected for further investigations. The familial co-segregation analysis sustained the significance of the identified gene alteration, and revealed an inheritance fashion of the mutation as both parents were found to be heterozygous carriers of the alteration (Table 2).

This identified novel CYP27B1 alteration displayed a deleterious nature and it is the likely cause of our patient’s clinical findings. This is implemented by: Firstly, the premature protein truncation effect of the alteration. The *c.1510C* > T(p.Q504X) alteration results from a C to T nucleotide substitution at position 1510. This changes an amino acid from a glutamine to a stop codon; and premature stop codons are typically deleterious in nature [9–11]. Secondly, the inconsistency of the alteration type with the previously reported predominant CYP27B1 alterations. Based on data search in the Human Gene Mutation Database (HGMD), 63 CYP27B1 alterations were reported: only 28.6% are protein truncating (5 nonsense, 13 frameshift insertions/deletions, 0 gross deletions), while 61.9% are non-truncating (38 missense, 1 small in-frame insertions/deletion), and 9.5% are possible protein-truncating (5 splice, 1 regulatory) [12]. Thirdly, the inheritance pattern of the detected alteration in our patient is consistent with an autosomal recessive fashion.

## Conclusion

The deleterious nature of the reported novel CYP27B1: *c.1510C > T*(p.Q504X) alteration and its inheritance fashion in our patient supports its classification as a pathogenic mutation for VDDR-1.

## Consent

Ethical approval for this study was obtained from the ethics committee at King Khalid University Hospital, and parents consented to genetic testing and consented to genetic testing in their child. Copies of the written consents are available for review by the Editor-in-Chief of this journal.

## Authors’ information

All of the authors in this article are currently employed by King Khalid University Hospital and King Saud University, Saudi Arabia. Amir Babiker (AB) is a Fellow of the Royal College of Paediatrics and Child Health. He is a Consultant Paediatric endocrinologist and Assistant Professor of Paediatrics. Iman Al Gadi (AG) is a Paediatric resident and demonstrator in Endocrine division (Paediatrics). Nasir A. Al Jurayyan (NAA), MD, is Professor of Paediatrics, Consultant Paediatric endocrinologist and Head of Endocrine division. Abdulrahman Al-Nemri (AA), MD, is Chairman of the Paediatric Department and Associate Professor of Paediatrics. Ali Abdu Haboob (AAA), MD, is a consultant Paediatric Intensivist and Assistant Professor of Paediatrics. Ahmed Amer Al Boukai (ABO), MD, FRKSU is an Associate Professor and Consultant Radiologist. Ali Al Zahrani (AA), a Technician in the Department of Pathology. Hanan Habib (HH), MD, is Professor of Pathology and a Consultant Pathologist.

## Abbreviations

1: 25(OH)_2_D3: 1,25 dihydroxyvitamin D3
25OHD3: 25 hydroxy vitamin D3
HGMD: Human Gene Mutation Database
PTH: Parathyroid hormone
VDDR-1: Vitamin D dependent rickets type 1
VDR: Vitamin D receptor
VDRR: Vitamin D resistant rickets
VDDR II: Vitamin D dependent rickets type II.
